# Cancer follow-up supported by patient-reported outcomes in patients undergoing intended curative complex surgery for advanced cancer

**DOI:** 10.1186/s41687-021-00391-1

**Published:** 2021-11-08

**Authors:** Sissel Ravn, Henriette Vind Thaysen, Victor Jilbert Verwaal, HIPEC ePRO collaborative at Aarhus University Hospital, Mette Møller Soerensen, Jonas Funder, Mette Shou Mikkelsen, Thora Christiansen, Charlotte Søgaard, Lene Seibæk, Lene Hjerrild Iversen

**Affiliations:** 1 Department of Surgery, Aarhus University Hospital, Palle Juul-Jensens Boulevard 99, 8200 Aarhus N, Denmark; 2 Department of Gynaecology and Obstetrics, Aarhus University Hospital, Aarhus, Denmark

**Keywords:** Patient involvement, Patient activation, Advanced cancer, Patient-reported outcomes, Follow-up

## Abstract

**Background and aim:**

Patient activation (PA) and Patient Involvement (PI) are considered elements in good survivorship. We aimed to evaluate the effect of a follow-up supported by electronic patient-reported outcomes (ePRO) on PA and PI.

**Method:**

From February 2017 to January 2019, we conducted an explorative interventional study. We included 187 patients followed after intended curative complex surgery for advanced cancer at two different Departments at a University Hospital. Prior to each follow-up consultation, patients used the ePRO to screen themselves for clinical important symptoms, function and needs. The ePRO was graphically presented to the clinician during the follow-up, aiming to facilitate patient activation and involvement in each follow-up. PA was measured by the Patient Activation Measurement (PAM), while PI was measured by five indicator questions. PAM and PI data compared between (− ePRO) and interventional (+ ePRO) consultations. PAM data were analysed using a linear mixed effect regression model with intervention (yes/no) and time along with the interaction between them as categorical fixed effects. The analyses were further adjusted for time (days) since surgery.

**Results:**

According to our data, ePRO supported consultations did not improve PA. The average mean difference in PAM score between + ePRO and − ePRO consultations were − 0.2 (95% confidence interval − 2.6; 2.2, p = 0.9). There was no statistically significant improvement in PAM scores over time in neither + ePRO nor − ePRO group (p = 0.5). Based on the five PI-indicator questions, the majority of all consultations were evaluated as “some, much or very much” involved in consultation; providing a wider scope of dialogue, encouraged patients to ask questions and share their experiences and concerns. Nevertheless, another few patients reported not to be involved at all in the consultations.

**Conclusion:**

We did not demonstrate evidence for ePRO supported consultations to improve patient activation, and patient activation did not improve over time. Our results generate the hypotheses that factors related to ePRO supported consultation had the potential to support PI by offering a wider scope of dialogue, and encourage patients to ask questions and share their experiences and concerns during follow-up.

## Introduction

Patients with metastases to the peritoneal surface have historically been treated with palliative intent receiving either systemic chemotherapy with or without symptom-directed surgery, or supportive treatment only, depending on their overall health performance [1–3]. With the introduction of intended curative complex cancer surgery (i.e. Cytoreductive Surgery (CRS) and Hyperthermic Intraperitoneal Chemotherapy (HIPEC), the prognosis for these patients has improved significantly [4–6]. Along with improved survival, survivorship issues have therefore become increasingly important during the postoperative follow-up [7]. The concept of CRS + HIPEC consists of major abdominal surgery with removal of all macroscopic tumour tissue, which includes stripping of tumour-involved parietal peritoneum and resection of infiltrated organs [8]. Finally, the abdomen is flushed with hyperthermic intraperitoneal chemotherapy [4].

Survivorship is defined as the “health and well-being of a person with cancer from the time of diagnosis until the end of life” [9]. A good Health-related Quality of Life (HRQoL) with decreased symptom distress [7] are key elements in this effort. To ensure this, a more patient-centred approach has been suggested [10, 11]. Patient-centered care is defined as a clinical practice that is respectful of and responsive to the patient’s preferences, needs and values [12]. The concept builds on the same values as patient involvement (PI), referring “specifically to the rights and benefits of patients to have a central position in the healthcare process, supporting patient activation, too [13–15].

The concept of patient activation (PA) can be defined as the patients’ individual level of knowledge, confidence and skills to manage their own health [16]. A number of studies have indicated that active patients are able to participate in follow-up, raise questions, make requests, state preferences and introduce topics [17, 18]. It has been demonstrated that an increase in patients’ activation is associated with a positive change in general health and lower health care costs [19, 20]. PA can be influenced by self-management strategies, and the use of patient-reported outcomes (PRO) in the consultations has been suggested as a tool to facilitate patient involvement and support PA [21, 22].

In the management of patients with metastases to the peritoneal surface, the primary focus has up till now been on the surgical treatment, morbidity, recurrence and survival [3, 23] and to a lesser extent the survivorship. Therefore, we conducted an explorative, interventional study with the aim to evaluate if a postoperative follow-up supported by electronic patient-reported outcomes (ePRO) was associated with increased level of PA and PI.

## Method

### Study design and setting

The study was carried out as an explorative interventional study in the period from February 2017 to January 2019 including patients with advanced cancer. In current study, advanced cancer was considered as metastases to the peritoneum originating from intraabdominal organs (i.e. colon, rectum, appendix, and ovaries) and pseudomyxoma peritonei with or without limited spread to organs such as liver or lungs. In case of metastases to the liver and/or lungs, all metastases were deemed treatable with curative intent). Patients with peritoneal metastases were treated with curative intent with complex cancer surgery at two different departments at Aarhus University Hospital. Both departments were national treatment centers for CRS + HIPEC. At Department of Surgery, the procedure was offered as standard treatment, whereas the treatment was performed as a part of a clinical trial at Department of Gynecology[24].

**Fig. 1:**
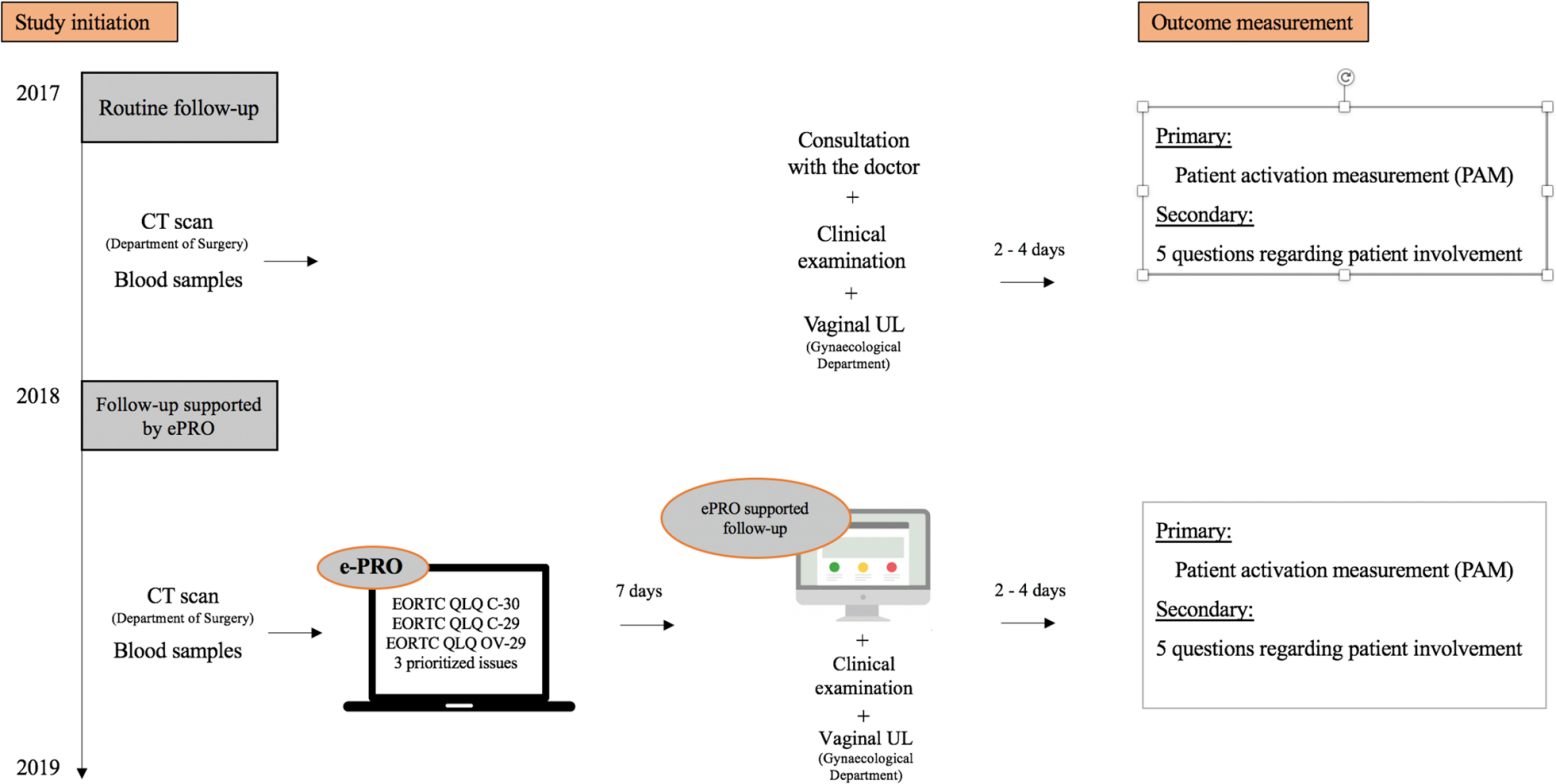
The setting. ePRO: electronic patient-reported outcomes, EORTC: European Organisation for Research and Treatment of Cancer

As depicted in Fig. 1, the routine follow-up was scheduled according to specific cancer disease of interest, and thus, unequal at the two departments.

At Department of Surgery, according to national guidelines, the routine follow-up included a consultation in the outpatient clinic at 3, 6, 12, 18, 24, 36, 48, and 60 months postoperatively. The standard follow-up included blood samples and a Computer Tomography (CT) of the thorax, abdomen and pelvis, with a subsequently physical follow-up visit containing results of the CT and a clinical examination.

At Department of Gynecology, the standard follow-up included blood samples (tumor marker), a physical follow-up with a clinical examination and a pelvic examination with a vaginal ultrasound. Imaging was only performed if recurrence was suspected.

### Participants

Patients from the two departments (Department of Surgery and Department of Gynecology) who had undergone CRS + HIPEC with curative intent were considered eligible for study inclusion. Inclusion were consecutively performed in the period from January 2017 to October 2019, irrespective of time since the complex surgery (i.e. patients could be included at any visit during the follow-up). Patients were included in the outpatient clinic or by telephone prior to each follow-up consultation. Informed written consent was signed on-site or sent by e-mail and returned either personally or by mail.

A follow-up for patients surviving peritoneal metastases can be dynamic due to several reasons. First, in the initial period after CRS + HIPC most patients receive systemic chemotherapy. Second, though beneficial survival effects have been demonstrated with CRS + HIPEC, recurrence still occur in a large part of the patients, which impacts the follow-up [25, 26].

Patients were not included in case of the following: (1) unable to speak and read Danish, (2) the forthcoming consultation would be the last (i.e., 60 months postoperatively), (3) no digital e-mail solution reached by public authorities and/or e-mail, (4) informed of recurrence at the consultation subjected to inclusion and (5) in a diagnostic process of recurrence.

### Intervention

An ePRO supported consultation was considered as the intervention. The ePRO included the validated questionnaires The European Organization for Research and Treatment of Cancer (EORTC) quality of life questionnaire (QlQ) C30 [27], C29 [28] and OV28 [29], and item 6 and 11 from the Hospital and Anxiety Depression scale [30]. Further, the ePRO provided opportunity for patients to state three concerns to be prioritized in the consultation.

The ePRO supported consultations were performed in the period from February 2018 to January 2019. The ePRO was sent out to the patient one week prior to a follow-up consultation. Each patient response was flagged with colors illustrating the severity according to the original response algorithm developed for each questionnaire [31]. The patient’s ePRO response was graphically presented to the clinician. After the consultation, clinicians were required to document the use of the e-PRO, either technically in the electronic system or with a comment in the Electronic Medical Record. All clinicians were provided with a one-page manual of how to prepare for, undergo and document an e-PRO-based consultation, supplied by a one-hour training session.

The ePRO was developed in collaboration with Ambuflex [32], which is an electronic system that uses PRO measures as the basis of follow-up to improve quality of care. Furthermore, the ePRO was developed and tested in co-operation between a small selected group of patients and clinicians. The development of the e-PRO is described in details elsewhere [31].

### Outcomes measurements

The primary outcome was PA and PI. These outcomes were evaluated with an electronic questionnaire (i.e. evaluation) sent out 2–4 days after each follow-up consultation to all study patients, before and during the intervention (Fig. 1).

PA was measured by the Danish validated 13-item Patient Activation measurement (PAM) questionnaire[33], which was developed and validated by Hibbard et al. to evaluate the patient’s ability to self-manage [16, 34]. The PAM scores from 0 to 100, where a higher score indicates a higher level of activation.

PI was described by five questions, which validity and reliability of these questions as indicators of PI had been tested among 3000 Danish patients [35]. The questions were as followed:

(I) The health care provider asked about my own experiences with my illness / condition
(II) I talked to the health care provider about the questions or concerns I had
(III) The health care professional encouraged me to ask questions or talk about concerns
(IV) I was on advice when deciding what was to happen
(V) I have had appropriate conversations with healthcare professionals about how to best manage my illness/condition.

Patients had following response categories “Not at all”, “Less”, “Some”, “Much” and “very much”.

### Statistical methods

Apart from the disease characteristics, which were retrieved from a local database, patient characteristics were collected with an online questionnaire at inclusion. Patient and disease characteristics are presented as frequencies and percentages for categorical variables, while continuous variables are presented as median with ranges. Patient characteristics are described according to following grouping: Patients only completed routine follow-up without ePRO were referred to as ‘− ePRO’. Patients who completed a routine follow-up (− ePRO) *and* an ePRO supported follow-up were referred to as ‘− /+ ePRO’. Patients who participated in an interventional follow-up with ePRO consultations were referred to as the ‘+ ePRO group’.

The PAM score ranges from 0 to 100 introducing ceiling and floor effects that affect the normal distribution of data. Currently, no guidelines exist on the presentation of PAM, and PAM is often presented as means with 95% confidence intervals despite its distribution. The Danish validated version of the 13-item PAM does not recommend the presentation of four levels [33], thus we restrained from this. Raw PAM scores were presented as group means with 95% confidence intervals. The PAM-score was analysed using a linear mixed effect regression model with ePRO as an intervention (yes/no) and time since surgery along with the interaction between them as categorical fixed effects. Patient was included as a random effect. In order to take into account that some patients had observations corresponding to both interventions, also a random treatment within patient effect was included. Model validation was performed by comparing observed and expected within subject standard deviations and correlations and by inspecting QQ-plots.

PI data were presented according to ePRO as an intervention (yes/no), yet multiple responses for each patient could occur in both groups (− ePRO/+ ePRO).

As this type of data can be analysed in several ways, we have presented them in a simple method as well. A mean PAM score was estimated for each patient, and presented according to group − ePRO, + ePRO and ‘− /+ ePRO.

All analyses were performed as complete case analyses, thus only patients who answered outcome measurements were included in the analysis. The statistical analyses were performed using STATA statistical software (STATA, release IC15, STATACorp, Texas, USA).

## Results

**Fig. 2:**
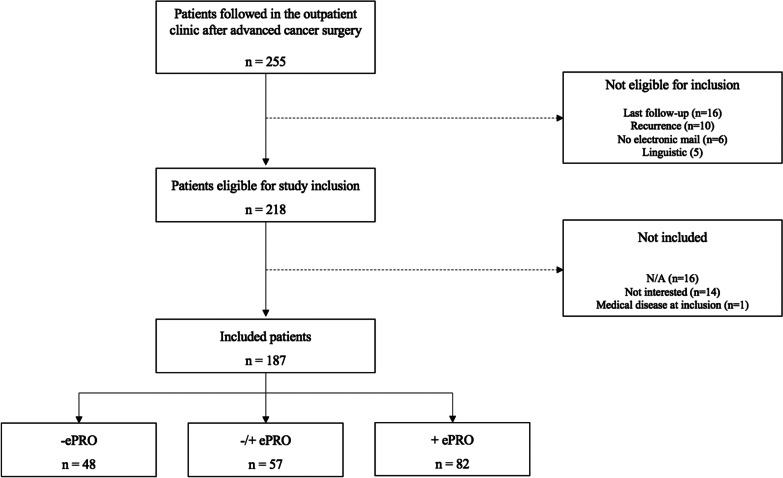
Inclusion of patients throughout the period from 2017 to 2018 (N/A: not available)

**Fig. 3:**
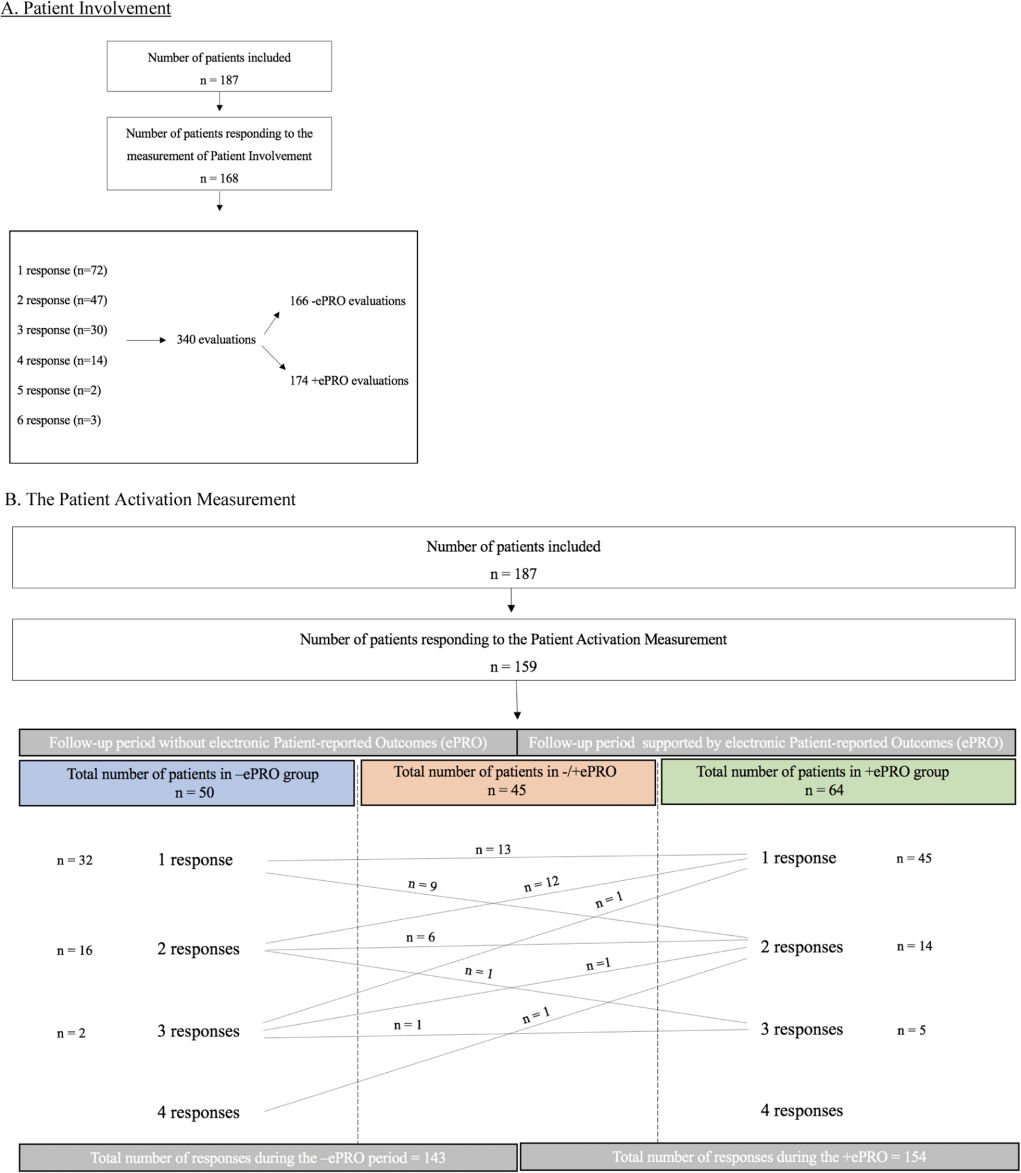
Demonstrates the number of patients included into the study (n = 187), and the number of patients who responded to the evaluation, e.g. measurements of patient involvement (**a**) (n = 168) and Patient Activation Measurements (**b**) (n = 159). Both **a** and **b** demonstrates the number of responses for each patient in the different follow-up periods. The routine follow-up without electronic Patient-reported Outcomes (ePRO) is referred to as − ePRO, whereas the follow-up period supported by electronic Patient-reported Outcomes (ePRO) are referred to as + ePRO. In **b** the lines demonstrate the number of responses each patient had in the − ePRO follow-up and the + ePRO follow-up

In total, 255 patients were followed in the outpatient clinic in the study period from 2017–2019. Among these, 218 patients were eligible for inclusion, and 187 (86%) patients accepted participation in the study (Fig. 2). Numbers of responses are shown in Fig. 3.

Baseline characteristics are presented according to the groups (− ePRO, + ePRO and − /+ ePRO), and summed in Table 1. Overall, the majority of patients were females, aged < 65 years, and a large part of the patients had PM originating from a gastrointestinal location (colorectal cancer and pseudomyxoma peritonei). In total, around 75% of the study population were in a relationship/married. The level of education was equally distributed between groups (− ePRO, + ePRO and − /+ ePRO), with nearly 50% having 2–4 years additional education in each group. At least 50% of patients in each group were not attached to the labor market (senior citizens / sick leave).

**Table 1:** Baseline characteristics presented for each group (− ePRO, − / + ePRO and + ePRO) and the total population. Groups are based on the type of follow-up. Patient and disease characteristics are presented as frequencies and percentages

Variable	Groups according to type of follow-up *Total: n* = *187*			
	− ePRO *n* = *48*	− /+ ePRO *n* = *57*	+ ePRO *n* = *82*	Total n = 187
Sex				
Female Male	35 (73) 13 (27)	32 (56) 25 (44)	44 (54) 38 (47)	111 (59) 76 (41)
Age (median, range)	57 (28–76)	61 (39–77)	59 (26–75)	59 (26–77)
Age				
< 60 60–65 65–70 > 70	29 (60) 6 (13) 10 (21) 3 (6)	23 (40) 11 (19) 14 (25) 9 (16)	42 (51) 10 (12) 19 (23) 11 (13)	94 (50) 27 (14) 43 (23) 23 (12)
Disease				
Pseudomyxoma peritonei Colorectal cancer Ovarian Malignant mesothelioma	13 (27) 28 (59) 4 (8) 3 (6)	20 (35) 31 (54) 4 (7) 2 (4)	21 (26) 49 (60) 10 (12) 2 (2)	54 (29) 108 (58) 18 (10) 7 (4)
Civil status				
Married/relationship Divorced/single Other Missing	39 (81) 7 (15) 0 2 (4)	47 (82) 9 (16) 0 1 (2)	54 (67) 11 (14) 1 (1) 15 (18)	141 (75) 27 (14) 1 (1) 18 (10)
Education				
Primary school High school/training + 2–4 years education + > 4 years education Missing	4 (8) 10 (21) 24 (50) 8 (17) 2 (4)	10 (18) 8 (14) 33 (58) 5 (9) 1 (2)	10 (12) 10 (12) 37 (45) 10 (12) 15 (18)	24 (13) 28 (15) 94 (50) 23 (12) 18 (10)
Labor				
Full-time Reduced time Senior citizen Sick leave Unemployed Unknown/missing	10 (21) 7 (15) 13 (27) 12 (25) 1 (2) 5 (10)	11 (19) 8 (14) 27 (47) 10 (18) 0 (0) 1 (2)	14 (17) 12 (15) 24 (29) 16 (20) 0 (0) 15 (18)	35 (19) 27 (14) 64 (34) 38 (20) 1 (1) 22 (12)

### Patient activation measurement

As demonstrated in Fig. 3, 159/187 patients responded to the evaluation resulting in 297 responses.

**Fig. 4:**
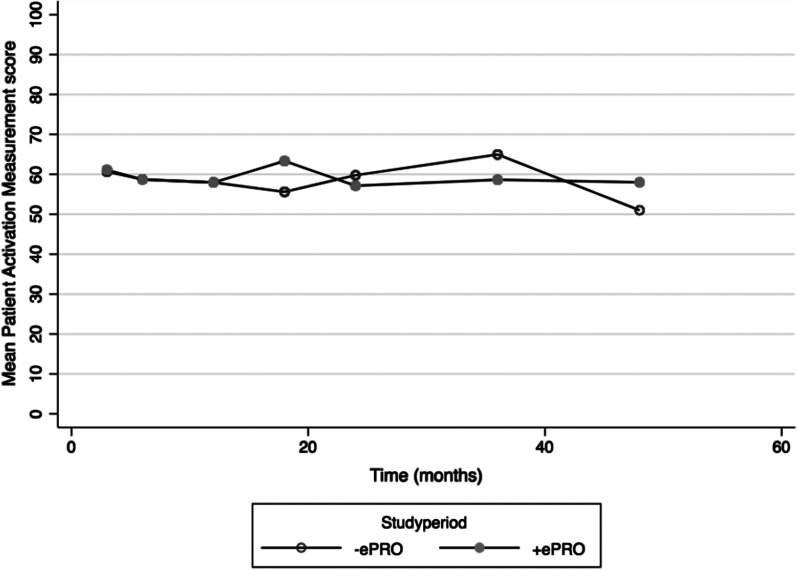
Raw Patient Activation Measurement (PAM) scores assessed after each follow-up consultation, i.e. 3, 6, 12, 18, 24, 36 and 48 months postoperatively. Each response is grouped according to the period with no electronic Patient-reported Outcomes (− ePRO) and the period supported by electronic Patient-reported Outcomes (+ ePRO)

Raw PAM mean scores are presented in Table 2 and Fig. 4. The linear mixed effect regression model did not show a statistically significant benefit of ePRO supported consultations. The average mean difference in PAM score between + ePRO and − ePRO consultations were − 0.2 (95% confidence interval − 2.6; 2.2, p = 0.9). There was no statistically significant improvement in PAM scores over time in neither + ePRO nor − ePRO group (p = 0.5).

**Table 2:** Raw patient activation measurement (PAM) scores with 95% confidence intervals (CI) assessed after each follow-up consultation, i.e. 3, 6, 12, 18, 24, 36 and 48 months postoperatively. Each response is grouped according to the period with no electronic Patient-reported Outcomes (− ePRO) and the period supported by electronic Patient-reported Outcomes (+ ePRO)

	Groups	
	− ePRO n = 105	+ ePRO n = 82
3 months	60.7 (57.5, 63.9) (n = 41)	61.1 (57.4, 64.8) (n = 27)
6 months	58.7 (53.9, 63.6) (n = 23)	58.7 (53.8, 63.6) (n = 28)
12 months	58.9 (54.2, 61.7) (n = 38)	58.0 (54.7, 61.2) (n = 36)
18 months	55.6 (51.0, 60.2) (n = 20)	63.3 (56.4, 70.3) (n = 20)
24 months	59.8 (52.4, 67.1) (n = 8)	58.6 (50.4, 63.9) (n = 17)
36 months	65.0 (56.7, 73.3) (n = 12)	58.6 (51.5, 65.8) (n = 17)
48 months	51 (NA) (n = 1)	58 (43.9, 72.1) (n = 9)

Presented with a simple method; the mean PAM score for patients with a routine follow-up (− ePRO) was 57.7 (55.5; 60.0) (n = 48), compared to 58.0 (55.5; 60.4) (n = 82) for patients with only ePRO supported consultations.

For patients with who completed both − ePRO and + ePRO consultations, the mean PAM score for − ePRO consultations was 60.6 (58.6; 62.6) (n = 57) compared to 60.3 (58.0; 62.5) (n = 57) for + ePRO consultations.

### Patient involvement

**Fig. 5:**
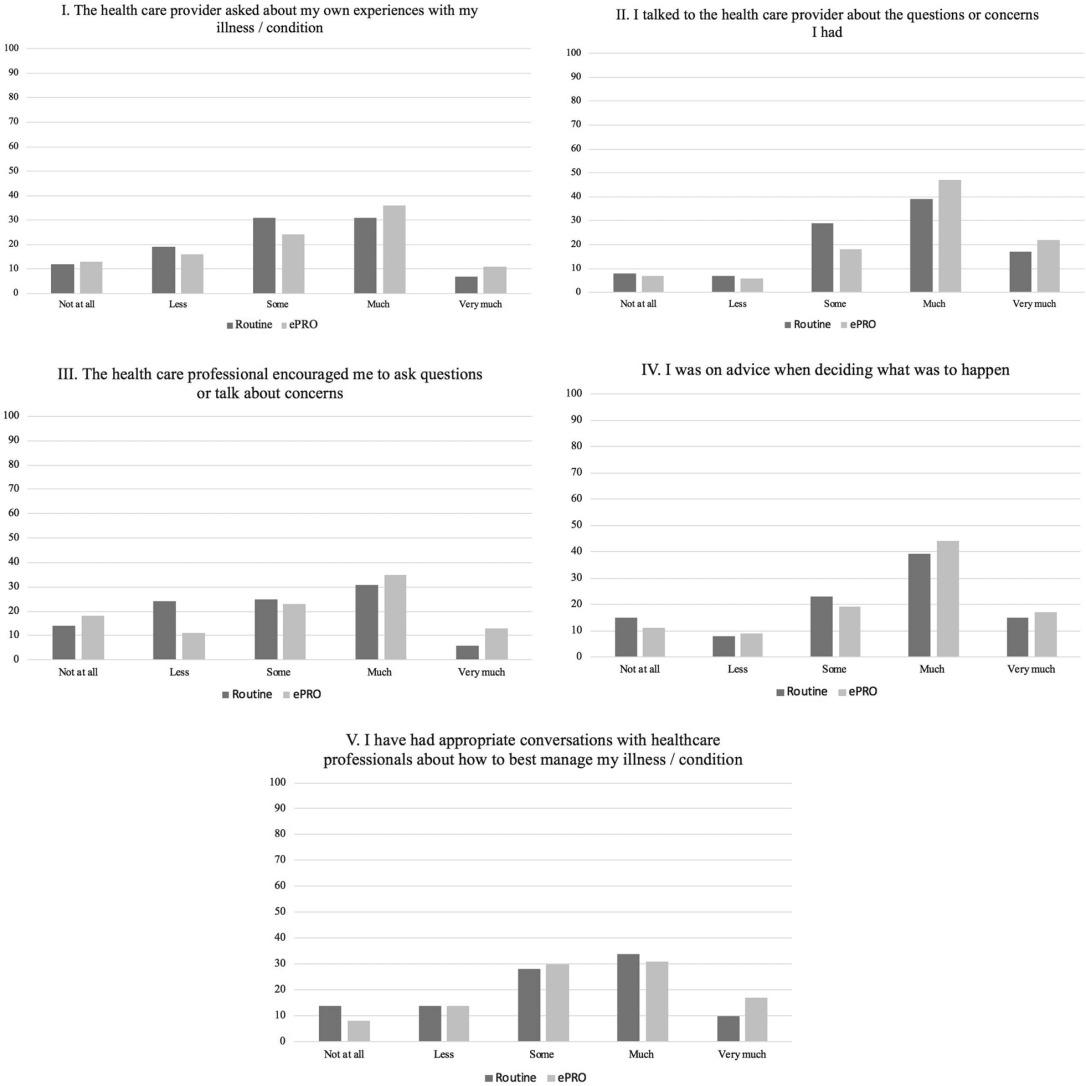
Patient’s assessment of patient involvement in the follow-up consultation stratified by − ePRO consultations and + e-PRO consultations

Patients' assessment of PI in the consultation is presented in Fig. 5. Overall, the majority of all consultations were assessed as ‘some’ or ‘much’ involved in the consultation. A large proportion of + ePRO consultations were assessed as ‘much’ or ‘very much’ involving.

## Discussion

We performed an explorative interventional study, aiming to increase PA among patients treated with curative intended complex cancer surgery. This study is important as it focus on survivorship in patients surviving advanced cancer. We implemented a patient-centered follow-up supported by electronic patient-reported outcomes (ePRO) to improve PA. Follow-up consultations supported by ePRO did not change PA.

### Patient involvement

Our results showed that the majority of all consultations were evaluated to have some, much or very much patient involvement. Regarding question I–III (I. *The health care provider asked about my own experiences with my illness/condition*, II. *I talked to the health care provider about the questions or concerns I had* and III. *The health care professional encouraged me to ask questions or talk about concerns*) a large proportion of + ePRO consultations were assessed as ‘much’ og ‘very much’ involving. The answers to these questions (I-III) may indicate that the use of ePRO provide a wider scope of dialogue and encourage patients to ask questions and share their experiences and concerns during follow-up consultations, which is also reported from other studies [21, 36]. However, it must be interpreted with caution due to multiple responses from each patient. Nevertheless, another few patients reported not to be involved at all when using ePRO in the consultations.

### Patient activation

In current study, we did not demonstrate an effect of ePRO supported consultations on PA. This might be due to several factors, which has previously been described, e.g. contextual factors and multiple patient and clinician characteristics [37].

Several contextual factors may increase the complexity of the implementation of ePRO, thus impact its effect on PA. Due to ethical considerations, the intervention was made to meet the requirements of the already existing follow-up program. Therefore, the intervention was confined to the specific times of follow-up for each patient (i.e. consecutive inclusion at follow-up times at 3, 6, 12, 18, 24, 48 and 36 months) why 3 months, as a minimum, existed between each interventional follow-up. This interval between the + ePRO consultations could potentially affect the patient’s level of activation, as learning strategies in both technical [38], and cognitive competences have the highest effect when executed intensively during a short period of time [33, 39]. However, our results did not demonstrate an increase in PA over time. In general, the implementation of a new set-up is affected by the existing organizational structure, and not automatically incorporated [40]. An organization must be capable of the development, integration and costs of structures that support technical solutions measuring and presenting health information [40]. In current setting, the ePRO was developed in collaboration with Ambuflex, which specializes in PRO as an electronic option. Therefore, the system was easily integrated in the Electronic Medical Record. Despite this being an electronic solution, the implementation of the ePRO, and the operation of the system and each patient was time consuming and managed manually by the first author. The impact of contextual factors remains unknown, and may be difficult to adjust for in any statistical analyses. In current study, it remains unknown if a change in contextual factors could have increased PA. In case of future implementation, organizations must earmark costs for the electronic solution and its everyday operation.

The PAM was primarily developed and reported in populations with chronic diseases (i.e. diabetes, ischemic heart disease, rheumatic diseases and asthma) [41], which differs from patients with advanced cancer. The lack of change in PA may be due to particular characteristics present in patients surgically treated for advanced cancer. It has been described that patients with peritoneal metastases experience severe preoperative mental pressure, affecting their ability to process health-care information in the peri-operative period[42]. Further, despite intended curative surgery, recurrence is frequent [25, 26]), and introduces fear. The impaired ability to process health-care information in combination with potential fear of recurrence might affect patient activation, since the patient’s ability to manage their health-care is dependent also on their emotional state [43]. On the other hand, the initial mean PAM measurements were high (PAM scores 60.7 and 61.1). Large changes in the PAM measurements have primarily been demonstrated in patients with initial low PAM scores [17, 44, 45]. Yet, in current study, follow-up consultations started at 3 months, and PAM scores did not change over time between groups. There may be several explanations to this. First, patients may already have accomplished sufficient knowledge, skills and confidence with respect to self-management [17]. Second, it might be the case that fewer clinical decisions are made during follow-up than during treatment, and the clinical decision is mostly performed by the clinicians due to the nature of the advanced cancer disease. Thus, patients have less influence on the consultations, and might explain why PA will not be affected by the use of ePRO.

It has previously been demonstrated that the use of PRO in clinical follow-up does not automatically enhance PI, and therefore the clinician’s role is important. In general, the clinician’s attitude towards PRO’s in a consultation (i.e. main component in current intervention) has been described as ambivalent [21, 46, 47], and highly depend on the clinicians’ day-to-day management of the system [48]. The lack of action from the health professionals to a problem reported by the patient in the PRO, induces unfilled patient expectations, and potentiates implementation barriers [21]. In current study, the clinician was responsible for the use and administration of the ePRO. Even though, data from the Ambuflex system could document whether or not the clinician looked at the ePRO, no data regarding the clinician’s use of the ePRO in the consultation were available. It has been suggested that the clinician’s attitude and management of (e)PRO is an important barrier for a feasible implementation of PRO in the clinic. Previous research has found that clinicians were ambivalent towards individualized follow-up based on PRO; some were positive and thought the PRO were beneficial, while others considered the PRO as a deterioration of the patient care and expressed suspicion regarding the value [48, 49]. Furthermore, has it been suggested that clinicians could find it difficult to respond and take action on the symptoms reported by the patient, and it has been recommended that to enable clinicians to manage PRO, training and preparation of the clinical staff members is necessary[50]. It remains unknown, wheatear or not the clinicians used the ePRO as intended, and to which extent it may have influenced on PA.

To summarize, an intervention with ePRO did not increase PA, which may be due to several factors; contextual factors, clinician’s role and patient characteristics (e.g. severe mental pressure and fear of recurrence). Hypothetically, the use of ePRO in itself is not sufficient to change self-management strategies.

### Strengths and limitations

Overall, our study is of explorative character, and results must be interpreted with caution. Even though we performed a linear mixed effect regression model to analyze our PAM data, some confounding factors cannot be controlled for. Furthermore, our PI results include multiple responses from some patients, which can introduce bias due to differences in response pattern between high-responders compared to low responders. Still, PI data were included as patient’s assessment of the PI level in each consultation was considered important. However, current study was not designed to perform comprehensive comparison between groups (i.e. − ePRO vs. + ePRO) of PI data.

Both the intervention and outcome were measured with validated PRO, yet never validated in such a cohort of patients undergoing complex surgery. Additionally, the ePRO was designed with the aim to improve PA and PI, however, since the development of the ePRO the general focus on survivorship and elements as patient involvement and patient activation has increased, and generated a lot of knowledge within the field. Clinicians’ knowledge and familiarity with PRO’s is important, and education/training may be needed to allow clinicians to utilize these instruments correctly and apply their data beneficially to their clinical practice. In this study, only a brief one-hour training session was provided [31]. We did not assess in which way, and to which extent the clinicians applied the ePRO. Finally, in current study patients were included at different times of follow-up, which potentially could affect the outcomes. However, we anticipated this by our adjusting for the time since surgery in the PAM analysis.

## Conclusion

We did not demonstrate evidence for ePRO supported consultations to improve patient activation. Our results generate the hypotheses that factors related to ePRO supported consultation had the potential to support PI by offering a wider scope of dialogue, and encourage patients to ask questions and share their experiences and concerns during follow-up. Further studies are needed.

## Data Availability

All data can be assessed by contacting the main author.

## Abbreviations

ePRO: Electronic patient-reported outcomes
EORTC: General European Organization for Research and Treatment of Cancer
PRO: Patient reported outcomes
PA: Patient activation
PI: Patient involvement
PAM: Patient activation measurement
